# Targeting Stromal-Cancer Cell Crosstalk Networks in Ovarian Cancer Treatment

**DOI:** 10.3390/biom6010003

**Published:** 2016-01-06

**Authors:** Tsz-Lun Yeung, Cecilia S. Leung, Fuhai Li, Stephen T. C. Wong, Samuel C. Mok

**Affiliations:** 1 Department of Gynecologic Oncology and Reproductive Medicine, The University of Texas MD Anderson Cancer Center, Houston, TX 77030, USA; TYeung@mdanderson.org (T.L.Y.); slleung@mdanderson.org (C.S.L.); 2 Department of Systems Medicine and Bioengineering, Houston Methodist Research Institute, Weill Cornell Medical College, Houston, TX 77030, USA; Fli@houstonmethodist.org (F.L.); STWong@houstonmethodist.org (S.S.T.W.); 3 National Cancer Institute Center for Modeling Cancer Development, Houston Methodist Research Institute, Houston, TX 77030, USA

**Keywords:** ovarian cancer, cancer-associated fibroblasts, stromal-tumor crosstalk, tumor microenvironment

## Abstract

Ovarian cancer is a histologically, clinically, and molecularly diverse disease with a five-year survival rate of less than 30%. It has been estimated that approximately 21,980 new cases of epithelial ovarian cancer will be diagnosed and 14,270 deaths will occur in the United States in 2015, making it the most lethal gynecologic malignancy. Ovarian tumor tissue is composed of cancer cells and a collection of different stromal cells. There is increasing evidence that demonstrates that stromal involvement is important in ovarian cancer pathogenesis. Therefore, stroma-specific signaling pathways, stroma-derived factors, and genetic changes in the tumor stroma present unique opportunities for improving the diagnosis and treatment of ovarian cancer. Cancer-associated fibroblasts (CAFs) are one of the major components of the tumor stroma that have demonstrated supportive roles in tumor progression. In this review, we highlight various types of signaling crosstalk between ovarian cancer cells and stromal cells, particularly with CAFs. In addition to evaluating the importance of signaling crosstalk in ovarian cancer progression, we discuss approaches that can be used to target tumor-promoting signaling crosstalk and how these approaches can be translated into potential ovarian cancer treatment.

## 1. Introduction

Ovarian cancer is one of the most lethal gynecological malignances and the fifth leading cause of cancer death in women in the United States [1]. Since most ovarian cancer patients are diagnosed at late stages, the overall survival rate remains a dismal 30%. Great research efforts have been put into facilitating the early detection of ovarian cancer; however, diagnosing patients at an early disease stage and beginning treatment before tumor progression and metastasis remains challenging because typical ovarian cancer symptoms, including abdominal discomfort, bloating, gas, nausea, and urinary urgency, can be subtle and are often ignored or mistaken for gastrointestinal problems [2,3].

The ovaries are made up of three major types of cells: epithelial cells, germ cells, and stromal cells. Each type of cell can develop into a different type of tumor. Among them, epithelial tumors are the most common ovarian tumors and can be histologically classified into the following four subtypes: (1) serous; (2) mucinous; (3) endometrioid; and (4) clear cell. Shih and Kurman proposed the classification of ovarian cancers on the basis of the pathways involved in tumorigenesis. In their proposed model, type I tumors are composed of low-grade serous, mucinous, endometrioid, and clear cell carcinomas and harbor genetic changes, including BRAF and KRAS mutations, that are rarely found in type II tumors. Type II tumors include high-grade serous carcinomas, which show frequent p53 mutations [4]. In recent years, gene expression profiles have been exploited to characterize and classify molecular subtypes of high-grade epithelial ovarian cancer [5]. In the study by Rea and colleagues, the authors analyzed the Axl, a receptor tyrosine kinase frequently overexpressed in solid tumors, -driven gene signature in 976 high-grade serous ovarian cancer patients and demonstrated the association of this gene signature to a group of patients with poor overall survival [6]. The focus of this review is on the high-grade serous subtype of epithelial ovarian cancer, which accounts for 70% of all ovarian cancer cases.

During ovarian tumor progression, metastasis often occurs throughout the peritoneal cavity. While multiple organs within the peritoneal cavity can be affected, ovarian cancer cells preferentially colonize the omentum during metastasis. In fact, omental metastases are observed in 80% of patients with serous ovarian cancer [7]. Ovarian cancer metastasis can occur via passive dissemination or hematogenous metastasis [8]. For ovarian cancer metastasis via passive dissemination, detached cancer cells from the primary tumor are carried by the peritoneal fluid to the peritoneum and omentum, where they attach to the substratum and establish metastatic tumor nodules. Passive dissemination of cancer cells has been accepted as the classic model and the most common route of ovarian cancer metastasis [9,10]. However, using a parabiosis animal model, researchers showed that ovarian cancer could metastasize through a hematogenous route [11]. In this model, ovarian tumor cells from the primary site enter the bloodstream by intravasation and are carried to the metastatic tumor site, where extravasation of the tumor cells and formation of the secondary tumor occur.

Ovarian cancer is notable for its initial sensitivity to chemotherapy. However, in most patients, the disease recurs as chemoresistant tumors within 12 to 24 months after initial diagnosis. Without effective treatment options, these patients often die of progressively chemotherapy-resistant disease. To improve treatment effectiveness and the survival of ovarian cancer patients, we need new therapeutic targets. While most current treatment options and therapeutic agents mainly target ovarian cancer cells, the importance of the tumor-supportive microenvironment must not be overlooked. In this review, we highlight different types of targetable signaling crosstalk between ovarian cancer cells and its tumor-stromal microenvironment, particularly the interactions between cancer cells and cancer-associated fibroblasts (CAFs). We also describe how such signaling crosstalk can be served as targets of novel therapeutic regimens for ovarian cancer.

## 2. Current Standards of Care for Ovarian Cancer

The conventional ovarian cancer treatment strategy involves debulking surgery, in which visible tumor nodules are removed before chemotherapy. Following cytoreductive surgery, ovarian cancer patients undergo platinum- and taxane-based chemotherapy. This treatment strategy is considered effective in patients whose cancer is detected and diagnosed at an early stage, when metastasis has yet to occur and the tumor mass is confined to the ovaries (stage I). With the combination of cytoreductive surgery and chemotherapy, the cure rate for early-stage ovarian cancer patients is as high as 90%. In addition to conventional cytoreductive surgery, neoadjuvant chemotherapy, in which patients receive chemotherapy before undergoing surgery to remove the tumor mass, is also used as a treatment strategy.

Recent research efforts have focused on the development and validation of a histopathological scoring system for measuring response to neoadjuvant chemotherapy in advanced-stage ovarian cancer patients [12]. Gorodnova and colleagues recently showed that ovarian cancer patients with a germ-line BRCA mutation demonstrated a high response rate to neoadjuvant chemotherapy; a complete response was observed in 34% of mutation carriers *vs.* 4% of non-carriers [13], suggesting that this treatment has greater clinical benefits in certain groups of patients. However, over 70% of ovarian cancer cases are diagnosed at an advanced stage, when cancer cells have already metastasized to other pelvic organs, including the bladder and uterus for stage II diseases, the abdomen for stage III diseases, and beyond the peritoneal cavity for stage IV diseases. In advanced-stage disease, cytoreductive surgery is less effective and optimal debulking is difficult to achieve [14,15]; thus, the cure rates in these patients decrease substantially.

## 3. Ovarian Tumor Microenvironment

To improve treatment effectiveness and the survival of ovarian cancer patients, new therapeutic targets (and, thus, new treatment regimens) are urgently needed. Most current treatment options and therapeutic agents target ovarian cancer cells and often overlook the importance of the tumor-supportive microenvironment. The tumor microenvironment, composed primarily of stromal fibroblasts, endothelial cells, immune cells, and extracellular matrix proteins derived from various cell types, can directly affect the phenotypes of cancer cells [16], thereby presenting a unique aspect of diagnosing and treating cancer. In ovarian cancer, the tumor stroma contributes to 7%–83% of the tumor tissue, with a median relative proportion of 50% [17]. The major cell types in the tumor microenvironment that support tumor progression include CAFs, certain immune cell types, endothelial cells, and cancer-associated adipocytes. Each of these cell types interacts with cancer cells, the extracellular matrix, and one another, contributing to the tumor-supportive microenvironment.

**Endothelial cells.** To progress and metastasize, ovarian cancer tissue must have sufficient tumor vasculature to obtain nutrients through circulation and to remove metabolic waste. In addition, the tumor vasculature is the major gateway for tumor cells to metastasize via the hematogenous route. In a variety of cancer types, including bladder cancer, lymphoma, multiple myeloma, breast cancer, and ovarian cancer, microvessel density is a poor prognostic marker [18,19,20,21,22,23], suggesting that tumor angiogenesis is important in disease progression. Cancer cell-derived pro-angiogenic protein vascular endothelial growth factor (VEGF) is associated with ascites formation in ovarian cancer patients and is an independent predictor for patient survival. In addition, the pre-treatment VEGF level demonstrated a direct association with the CA125 level after three cycles of platinum-based chemotherapy, suggesting that it can be used as a predictive indicator for refractoriness to chemotherapy [24]. Recently, Slamakpour-Reihani and colleagues performed a study of the prognostic significance of angiogenic gene expression in serous ovarian cancer patients. Thirty-one angiogenesis-related genes were shown to be significantly associated with patient survival and were independently validated using datasets deposited in TCGA or NIH Gene Expression Omnibus [25]. These research findings suggest that endothelial cells, the basic building blocks of the tumor vasculature, can significantly impact tumor growth, metastasis, and response to chemotherapy in response to angiogenic factors produced by cancer cells and other stromal cell types.

**Adipocytes.** When ovarian cancer metastasizes, via passive dissemination of cancer cells from the primary site or the hematogenous route through blood vessels, the omentum is the preferred site for the establishment of metastatic tumor nodules [7,8]. The omentum is a piece of peritoneal fold that is composed primarily of adipocytes. These adipocytes, which are in close proximity to ovarian cancer cells, are termed cancer-associated adipocytes [26]. Adipose tissue is known to affect tumor development and progression. In some cancers, including endometrial and renal cancer, obesity is a risk factor that is associated with an increased incidence of cancer development [27]. In ovarian cancer, obesity and body mass index are generally recognized as insignificantly associated with cancer incidence and mortality [27,28,29]. However, Kato and colleagues recently reported that, in an assessment of an ovarian cancer patient cohort that consisted of 33 healthy weight and 37 overweight individuals, as well as an external cohort from the TCGA database, both overall and progression-free survival rates were significantly lower in overweight patients [30]. Therefore, the scientific community should revisit the linkage between obesity and ovarian cancer.

It is believed that ovarian cancer-associated adipocytes promote cancer progression through local interactions with cancer cells within the tumor microenvironment [27]. Studies have shown that cancer-associated adipocytes and adipose-derived mesenchymal stem cells, from which cancer-associated adipocytes are derived, promote ovarian cancer cell proliferation, motility, and resistance to chemotherapeutic agents [31,32,33,34,35,36]. Cancer-associated adipocytes secrete cytokines into the surrounding microenvironment [37]. These adipose tissue-derived cytokines, termed adipokines, support tumor progression. Among them, leptin was the first identified adipokine. Leptin has been shown to stimulate ovarian cancer cell migration and invasion potential through the activation of JAK/Stat3, PI3K/Akt, and RhoA/ROCK signaling in cancer cells and subsequent stress fiber and focal adhesion formation. Leptin also facilitates the maintenance of stemness and the mesenchymal phenotype of ovarian cancer cells [30,38]. In addition, leptin signaling in cancer cells has been shown to inhibit apoptosis and promote cell division by suppressing p21 and up-regulating cyclin D, which controls the G1/S cell cycle checkpoint [39,40]. Several other adipokines, including TNF-α, IL-6, IL-8, and monocyte chemoattractant protein-1, also have important roles in tumor progression [41,42,43]. In addition, high IL-6 levels in ascites can be used to distinguish patients with advanced serous ovarian cancer from patients with benign conditions and to predict chemoresistance and progression-free survival in ovarian cancer patients [44].

Overall, in the ovarian tumor microenvironment, cancer-associated adipocytes serve as the source of both tumor-promoting adipokines and energy-rich fatty acids for cancer cells [7,45]. Nieman and colleagues showed that, in addition to the promotion of tumor metastasis by adipocyte-derived IL-6 and IL-8, adipocytes directly transferred fatty acids to cancer cells in a co-culture model that consisted of fluorescently labeled lipid-loaded adipocytes and ovarian cancer cells. Further co-culture study showed that adipocytes support ovarian cancer cell proliferation by providing cancer cells with energy-dense lipids and activating β-oxidation in cancer cells [7].

**Immune infiltrates.** In the tumor microenvironment, immune infiltrates such as cytotoxic T cells, regulatory T cells, and macrophages interact with each other and with cancer cells and play important roles in ovarian cancer progression. Cytotoxic T cells, also called killer T cells, are T lymphocytes that are capable of killing cancer cells. Cytotoxic T cells express surface glycoprotein CD8 and T cell receptors that recognize tumor-specific antigens presented through MHC molecules by antigen-presenting cells in the tumor tissue. Upon antigen-specific activation, IL-2 promotes the clonal expansion of cytotoxic T cells [46]. Activated cytotoxic T cells produce and release cytotoxins, including granzymes and perforin. Perforin binds to the cell membrane of cancer cells and leads to the formation of pores. Facilitated by pore formation on the cancer cell surface, serine proteases called granzymes enter cancer cells and induce apoptosis [47]. The high density of infiltrating CD8^+^ T cells is a favorable prognostic marker and is associated with higher overall survival rates in ovarian cancer [48,49]. However, secretory ligand programmed death ligand-1 (PD-L1), which is produced by various cell types in the tumor tissue, can inhibit cytotoxic T cell activation and prevent cancer cells from being eradicated by the immune system [50].

PD-L1, when engaged with its receptor, PD-1, on T cells, suppresses IL-2-mediated T cell proliferation. It has been shown that PD-L1 production is significantly up-regulated in monocytes in patients with malignant ovarian cancer [51], and PD-L1 knockdown in SKOV3 ovarian cancer cells sensitizes them to T cell killing [52]. Using a preclinical ovarian cancer model, Mony and colleagues demonstrated that anti-PD-L1 antibody triggered T cell infiltration into the tumor tissue and prolonged the survival of tumor-bearing animals [53]. Regulatory T cells (Tregs) are FOXP3-expressing T lymphocytes that exert suppressive effects on CD8^+^ cytotoxic T cell activation. In ovarian cancer, it has been shown that *in vitro*-induced Tregs inhibit naïve T cell proliferation via TGF-β and INF-γ [54]. In a study that analyzed a cohort of over 400 ovarian cancer patients, Knutson and colleagues showed that the ratio of CD8+ T cells to Tregs was associated with improved survival rates [55]. Omentum consists of a large number of infiltrated macrophages. These tumor-associated macrophages (TAMs), particularly M2 TAMs, have been shown to facilitate ovarian tumor progression [56,57]. Hagemann and colleagues also demonstrated that macrophages promote cancer cell invasiveness via activation of NF-κB and JNK signaling [58]. Ovarian tumor-associated macrophage derived proteins, including IL-6, IL-8, and VEGF-C, have been shown to promote cancer progression [59,60]. Ko and colleagues recently reported that the homeobox gene HOXA9, via peritoneal M2 TAMs, stimulates ovarian cancer progression by promoting an immunosuppressive microenvironment [61]. Hence, targeting signaling crosstalk between ovarian cancer cells and cancer-associated immune cells could open up to a new vista for effective cancer therapy.

## 4. Cancer-Associated Fibroblasts and Their Crosstalk with Ovarian Cancer Cells

**CAFs and tumor progression.** Fibroblasts are one of the primary stromal cell types in ovarian tumor tissues [62]. In the 1970s, it was noticed that fibroblasts within tumor stroma acquired phenotypes similar to those of fibroblasts associated with wound healing; these fibroblasts were named CAFs [63,64,65,66]. CAFs can originate from the transformation of normal local fibroblasts [67] or from homing of differentiated bone marrow-derived progenitor cells to the tumor site [62]. Common markers for CAFs include α-smooth muscle actin [68], stromal-derived factor 1α (SDF-1) [69], fibroblast activation protein-1α (FAPα) [62], and fibroblast-specific protein-1 [70].

Recently, Lawrenson and colleagues identified natriuretic peptide B (NPPB), a CAF-specific secretory protein, as a tumor biomarker for ovarian cancer. In their study, it was shown that NPPB was expressed in 60% of primary ovarian CAF tissues but not in normal ovarian stroma [71]. The identification of unique signaling crosstalk networks allows the reprogramming of activated CAFs back to their “normal state” and could provide us with novel and potentially more effective therapeutic targets with fewer treatment-associated side effects [72]. CAF is known to secrete CAF-specific proteins, cytokines, and growth factors and produce an extracellular matrix that supports tumor cell growth and angiogenesis [66,73,74,75]. Among the common CAF-derived factors, FAPα has been shown to promote ovarian cancer proliferation and invasion via the engagement of α3β1 integrin receptor and the up-regulation of the ERK signaling pathway in ovarian cancer cells [76,77]. Besides FAPα, CAF-derived SDF-1, also known as CXCL12, has been shown to promote tumor growth, motility, and tumor angiogenesis in multiple cancer types, including ovarian cancer, by interacting with the CXCR4 receptors on cancer cells [78,79,80,81,82]. In addition to the well-studied classic CAF-derived factors, Lau and colleagues recently reported reciprocal tumor-stroma interactions in ovarian cancer [83]. In their study, while ovarian cancer cells were not the major responsive cells for tumor-derived lymphotoxin, CAFs responded to lymphotoxin via lymphotoxin-β receptor and the NF-κB signaling pathway. Ovarian cancer cell-derived lymphotoxin up-regulates CXCL11 secretion by CAFs; CXCL11, on the other hand, activates chemokine receptor CXCR3 on ovarian cancer cells and promotes their proliferation and motility. In addition to interacting with tumor cells through CAF-derived secretory factors, the expression of adhesion molecules by stromal cells modulates the interactions between tumor cells and the microenvironment. McAndrews and colleagues showed that stromal fibroblasts are more adherent to invasive ovarian cancer cells when compared to non-invasive ovarian cancer cells. Such adhesion is mediated by cadherin 11 and cadherin 2, which are highly expressed by invasive cancer cells and CAFs [84].

**Current research challenges.** Increasing evidence shows that CAFs can modulate cancer phenotypes; thus, researchers are interested in identifying the protein factors that are exclusively produced by CAFs, as well as the signaling crosstalk that exist between ovarian cancer cells and CAFs. While a study of the protein factors and signaling crosstalk between the two cell types would shed light on our understanding of ovarian cancer pathogenesis and on the identification of new potential therapeutic targets, identifying the protein factors that are unique to CAFs or cancer cells and revealing cell type-specific signaling pathways remain challenging. This is partly because cell-cell interactions are complex and can involve multi-directional communication between two or more cell types within the tumor tissue. Transcriptome profiles of ovarian tumor tissues have been generated by multiple research teams, including a large-scale dataset by The Cancer Genome Atlas (TCGA) [85,86,87]; while these data are useful for identifying differentially-expressed genes, deregulated proteins, and specific mutations associated with clinical outcomes, revealing specific cell-cell interaction and molecular crosstalk between cell types can be difficult using expression profiles generated from bulk tumor tissues, which often contain varying amount of tumor stroma and immune infiltrates. Such limitations highlight the importance of using cell type-specific expression profiles for the identification of crosstalk signaling networks.

**Uncovering crosstalk networks through laser microdissection.** To identify CAF-specific protein factors that can modulate cancer progression, Yeung and colleagues performed laser microdissection (LCM) on ovarian tumor tissues from cancer patients [73]. Individual components in the tumor microenvironment were isolated and profiled separately. In addition to transcriptome profiles of CAFs and ovarian cancer cells, epithelial and stromal components from normal ovaries were also microdissected and profiled. This approach allowed the identification of a CAF-specific gene signature. Further studies revealed that the TGF-β-rich ovarian tumor microenvironment up-regulates the production of versican (VCAN), a large extracellular matrix proteoglycan, in CAFs. Increased secretion of VCAN by CAFs promotes ovarian cancer cell migration and invasion by activating their NF-κB signaling pathway and increasing the expression levels of the motility/invasion-related genes CD44, HMMR, and MMP9. Previous research findings suggest that TGF-β ligands have limited direct effects on ovarian cancer cells [88,89]. Using expression profiles from individual microdissected cell types, Yeung and colleagues found that ovarian cancer cells had a diminished response to the cytokine TGF-β due to down-regulated TGF-β receptor expression. On the contrary, TGF-β receptors in CAFs are up-regulated compared to those in normal ovarian fibroblasts. The identified TGF-β-mediated signaling crosstalk between CAFs and ovarian cancer cells suggested that CAFs play crucial roles in TGF-β-induced ovarian cancer progression. Targeting TGF-β signaling in CAFs could be developed into an impactful targeted therapy for ovarian cancer.

Leung and colleagues showed that another member of the microdissected CAF-specific gene signature, microfibrillar-associated protein 5 (MFAP5), is a prognostic marker of poor survival rates in patients with ovarian cancer. They also demonstrated the roles of MFAP5 in enhancing cancer cell motility and invasion potential in ovarian cancer and showed that the tumor-promoting roles of CAF-derived MFAP5 are mediated via the activation of the calcium-dependent FAK/CREB/troponin C type 1 (TNNC1) signaling pathway in ovarian cancer cells by engaging the α_V_β_3_ integrin receptors [90]. CAF-derived MFAP5 induces F-actin cytoskeleton rearrangement and stress fiber formation in ovarian cancer cells. *In vivo*, an intraovarian cancer cell injection model demonstrated that targeting stromal MFAP5 using siRNA delivered by nanoparticles significantly reduced tumor growth and metastasis [90], suggesting that targeting the CAF-derived secretory factor is a new treatment approach for ovarian cancer.

In addition to delineate the signaling mechanisms and functional roles of CAF-derived tumor promoting factors, the aforementioned study highlighted the potential use of LCM in uncovering complex cell-cell communication and signaling networks in human cancers. With cell type-specific markers available for labeling, LCM can efficiently isolate cell types of interest from tumor tissue sections. Through advancements in next-generation sequencing, transcriptome profiling, miRNA profiling, and mutation detection can be performed using only the small amount of samples obtained from LCM. In addition to CAFs, immunohistochemistry-guided microdissection of cancer-associated endothelial cells from ovarian cancer tissue was performed to identify tumor vascular markers [91,92]. A similar approach can be extended to the isolation and profiling of immune infiltrates in tumor tissue. With the vigorous development of cancer immunotherapy, it is exciting to generate expression profiles from different immune cell types and investigate their signaling crosstalk with cancer cells. Even though LCM remains relatively labor intensive compared to other cell isolation methods, including FACS sorting using magnetic beads coated with specific antibodies, LCM has the unique advantage of preserving histological information during cell identification and isolation. For example, it allows researchers to determine whether they are obtaining CAFs at the tumor-stroma invasion front or isolating activated T lymphocytes from an intratumoral region. Such information would be useful for data interpretation and association of the signaling networks identified with clinical observations.

**Computational modeling.** Even with cell type-specific transcriptome profiles generated from tissue samples from cancer patients, the identification of crosstalk signaling between cell types that impact disease progression systematically could be overwhelming and time consuming. Therefore, computational tools are being developed to assist the discovery of signaling crosstalk between cancer cells and stromal cells involved in disease progression using cell type-specific expression profiles. Choi and colleagues reported the development of such a computational model for crosstalk signaling discovery [93]. Using RNA sequencing data derived from the sorted cells in tissue samples of animal model and tumor patients, and through computational modeling on the RNA sequencing data and information from public protein and pathway databases with the Cell-Cell Communication Explorer (CCCExplorer), they identified and validated paracrine and autocrine crosstalk networks in non-small-cell lung cancer. This computational signaling crosstalk model exemplifies how the computational methods can be used to facilitate the analysis of complex stromal-tumor transcriptome datasets and uncover novel interactions between different types of cells in the tumor microenvironment. We exploited the CCCExplorer to analyze our microdissected ovarian cancer cell and CAF transcriptome profiles and identified new signaling crosstalk between the two cell types, besides those we previously reported. We demonstrated that TGF-β can activate Smad signaling in CAFs. Blocking TGF-β/Smad signaling in CAFs using TGF-β receptor inhibitors (ALK inhibitors) or Smad inhibitor SIS3 significantly inhibited the aggressive phenotypes of ovarian cancer cells in a co-culture model [73], suggesting that activation of Smad signaling in CAFs promotes tumor progression. A CCCExplorer analysis result confirmed the activation of Smad signaling in CAFs; in addition, it showed that Smad signaling in CAFs can be activated by several up-regulated cancer-derived ligands.

## 5. Approaches to Targeting Tumor-Stroma Signaling Networks

**Figure 1:**
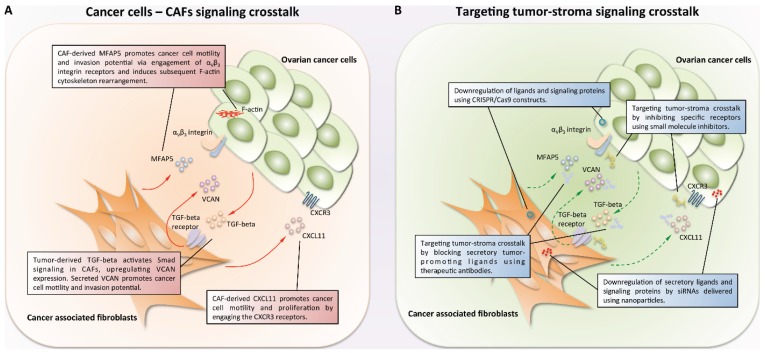
Signaling crosstalk and its targeting in ovarian cancer. (**A**) Crosstalks between ovarian cancer cells and stromal CAFs promote tumor progression. Here, we illustrated three signaling crosstalk events between ovarian cancer cells and cancer-associated fibroblasts (CAFs). In the TGF-β-rich ovarian tumor microenvironment, Smad signaling was induced in CAFs through activation of TGF-β receptors. This stimulus increases the production of the CAF-derived secretory protein VCAN. VCAN, when acts on cancer cells, activates the NF-κB signaling, and subsequently promotes migration and invasion of cancer cells via the up-regulation of motility/invasion-related genes CD44, HMMR, and MMP9. On the other hand, CAF-derived MFAP5 binds to the α_V_β_3_ integrin on the cancer cell and activates the calcium-dependent FAK/CREB/TNNC1 signaling pathways, which subsequently stimulate the reorganization of the F-actin cytoskeleton, thereby enhancing ovarian cancer cell motility. CAF-derived chemokine (C-X-C motif) ligand 11 (CXCL11) promotes cancer cell growth and migration via activation of the chemokine (C-X-C motif) receptor type 3 (CXCR3) on the cancer cell surface; and (**B**) multiple approaches can be used to target specific components within the cancer cell/CAF crosstalk signaling networks. Gene silencing can down-regulate the tumor- or stroma-derived secretory ligands that promote tumor progression by delivering specific gene-targeting siRNAs into tumor cells or CAFs using nanoparticles. Secretory factors from cancer cells and CAFs can be targeted using blocking antibodies. In addition, if specific receptors are involved in the crosstalk signaling, small molecule inhibitors can be used to inhibit the activation of these receptors. Lastly, gene editing using CRISPR/Cas9 technology can be developed into a therapeutic approach to down-regulate tumor- or stroma-derived secretory ligands and signaling molecules.

Targeting the stroma-specific signaling networks for cancer treatment has two benefits: (1) The ongoing functions of stromal cells are curial for the growth of cancer cells in the microenvironment; therefore, targeting these tumor-supportive stroma can inhibit tumor progression indirectly; and (2) stromal cells are often more genetically stable than are cancer cells; therefore, stromal cells, including CAFs, are less likely to accumulate adaptive mutations during treatment and acquire resistance to therapeutic agents [94,95,96]. Approaches that can be used to modulate specific therapeutic targets in the tumor-stroma signaling networks are shown in Figure 1.

**Nanotechnology.** Nanomedicine provides huge opportunities to improve current cancer treatment by facilitating targeted drug delivery and reducing systemic toxicity. Different types of nanoparticles, such as liposomes, polymeric nanoparticles, dendrimers, gold nanoparticles, carbon nanotubes, and quantum dots, are available for drug or gene delivery [97]. Due to the advantages of nanoparticles over conventional therapeutic regimens, several nanomedicine formulations have been approved by the FDA. For example, a PEGylated liposomal doxorubicin (Doxil) has been approved for the treatment of recurrent ovarian cancer, while a polyamino acid-bound paclitaxel, paclitaxel poliglumex, is being studied in a phase III clinical trial for ovarian cancer. Although most nanoparticle drug delivery systems rely on the passive accumulation of nanoparticles at the tumor sites through the enhanced permeability and retention effect, there are a plethora of ways to improve the specificity and efficiency of drug delivery by nanoparticles. One way to enhance tumor selectivity is to attach tumor-specific ligands (e.g., HER2 antibody, aptamers, and transferrin) to target signal transduction pathways in cancer. Other strategies have been developed to target the stromal populations. Chitosan nanoparticles have shown effectiveness in targeting CAF-derived tumorigenic factor, MFAP5, *in vivo* [90], and chitosan nanoparticles labeled with RGD peptides localize to the tumor vasculature and exert anti-angiogenic effects [98]. Moving forward, further research is required to achieve spatiotemporal control over drug release and resolve issues related to tumor heterogeneity, cost considerations, and nanoparticle stability after ligand conjugation.

**Antibodies.** The discovery that antibodies can be used as “magic bullets” for cancer diagnosis and treatment has been well established over the past decade, and antibody-based therapy is one of the most successful anti-tumor strategies [99]. Antibodies’ mechanisms of tumor cell killing include direct tumor cell killing via receptor agonist or antagonist activity, immune-mediated cell killing, payload delivery, and stromal cell inhibition [99]. Multiple -omics (e.g., genomics and proteomics) analyses have been performed to identify targetable antigens and receptors that drive cancer cell proliferation. Successful targets for antibody therapy include epidermal growth factor receptor (EGFR) [100], VEGF [101], PD-1 [102,103], and cytotoxic T lymphocyte-associated antigen 4 (CTLA-4) [104].

Despite the generally promising data on antibody-based therapy, it is crucial to balance the toxicity and therapeutic efficacy of antibody treatment. Biodistribution in patients and the uptake ratio in tumor *versus* normal tissues should be carefully determined to evaluate *in vivo* specificity. In ovarian cancer, antibody-based antiangiogenic therapies that target tumor-derived angiogenic factor using bevacizumab, a VEGF alpha-targeting monoclonal antibody, were extensively studied in multiple clinical trials to determine their therapeutic efficacy. However, despite the encouraging phase I and II trial results, phase III randomized trials of bevacizumab as front-line treatment and as treatment for recurrent ovarian cancer demonstrated only modest improvement in progression-free survival [105,106,107,108]. These results suggest that further discovery of pro-angiogenic factors that can be used as therapeutic targets, optimization of the treatment regimen, and the identification of therapy-sensitive patients are required to maximize the efficacy of therapeutic antibodies in cancer treatment.

Trastuzumab is a humanized monoclonal antibody that inhibits HER2-positive tumor growth by targeting the extracellular domain of HER2 receptor. Trastuzumab is currently being used as a targeted therapeutic agent for HER2-positive breast cancer. While approximately 25%–30% of breast cancer patients overexpress the HER2 receptor [109,110], clinical studies reported lower rates of HER2 overexpression in advanced ovarian cancer. In a phase II trial of the Gynecologic Oncology Group, Bookman and colleagues analyzed the HER2 expression levels in 837 tumor samples, and showed that 11.4% of these samples exhibited overexpression of the receptor. Among patents that demonstrated HER2 overexpression, 41 of them were enrolled to evaluate the clinical value of single-agent trastuzumab in recurrent ovarian cancer, and an overall response rate of 7.3% was observed [111]. In another study involving the screening of HER2 status of 320 ovarian cancer patients, Tueffred and colleagues reported that 6.6% of patients had HER2 overexpression [112]. Though the clinical value of single-agent trastuzumab seems to be limited by the low response rate in HER2 overexpressing ovarian cancer patients, researchers demonstrated that HER receptor expression by cancer cells may not give an accurate prediction on the responsiveness to HER-targeted therapeutic agents [113,114]. A study by Wilken and colleagues showed that trastuzumab sensitized ovarian cancer cells to EGFR-targeted agents. Ovarian cancer cells that are not growth suppressed by trastuzumab treatment are still responsive to the therapeutic agent and trastuzumab could be utilized as a primer for EGFR-targeted therapy [115]. To inhibit ovarian tumor progression by targeting adipocytes, Nieman and colleagues showed that antibodies that target omental adipocytes-derived IL-6, IL-8, MCP-1, and TIMP-1 significantly reduced homing of ovarian cancer cells toward adipocytes [7]. Taken together, while careful optimization is required, antibodies are considered effective and specific tools for targeting tumor-supporting stroma-derived secretory factors.

**Small molecule inhibitors.** Accelerated by cancer genome sequencing and RNA interference based screening, the discovery and development of small molecule cancer drugs has blossomed into a promising cancer therapeutic avenue [116]. Selective small molecule inhibitors for a plethora of kinases have been developed [117]. For example, the EGFR kinase inhibitors gefitinib and erlotinib for non-small cell lung cancer patients; the EGFR/ERBB2 inhibitor lapatinib for ERBB2-positive breast cancer patients, and the VEGFR kinase inhibitor sorafenib for patients with renal cancer [118]. The CYP171A1 inhibitor abiraterone, which blocks androgen synthesis, was approved for advanced-stage prostate cancer, and the BRAF inhibitor vemurafenib was approved for metastatic melanoma with the BRAF V600E mutation. However, bringing a new drug to market is costly and has an incredibly high rate of failure.

The complexity of genetic alterations and heterogeneity, both among different tumors and within the same tumor mass, makes the identification of key driver mutations and the corresponding therapies challenging [119,120,121]. In addition, many targets with significant disease linkage, such as mutated RAS proteins, c-MYC and hypoxia-inducible factor (HIF), are considered undruggable [122,123]. In ovarian cancer, small molecule inhibitor of the bone morphogenetic protein pathway, DMH1, inhibits tumor cell proliferation [124], and orally active small molecule inhibitors of gp130 and c-Met reduce tumor burden in mouse xenograft models [125,126]. In addition, metformin treatment on differentiated adipocytes inhibits adipogenesis and the subsequent ovarian tumor proliferation and migration via suppression of transcription factors CEBPα, CEBPβ, and SREBP1 [127]. Despite of being an effective approach to inhibit molecular targets, cells under prolonged treatment of small molecular inhibitors, often acquire resistance via selection for mutant alleles or up-regulation of alternative signaling pathways. Hence, the identification and development of multiple inhibitors that target different pathways are needed, as is the development of synergistic inhibitor combinations.

Computational approaches have been developed to facilitate the screening of small molecule drugs and to identify drug combinations. Huang and colleagues developed a systematic computational tool named DrugComboRanker that can be used to prioritize synergistic drug combinations and to better understand their mechanisms of action. After disease-specific signaling networks were identified, drug combinations were analyzed and prioritized by matching their targets with the disease signaling networks [128]. In addition to traditional high throughput screening using biochemistry or biology assays, both single drug and drug combination screening have recently benefited from the rapid development of high-throughput imaging-based assays [129,130,131]. Imaging-based high-content phenotypic screening of therapeutic agents allows the direct visualization and comparison of cellular phenotypes before and after drug treatment. Phenotypes visualized by automated digital microscopy include but are not limited to cell shape, motility, density, distribution, and cell cycle progression. Phenotypic information obtained through automated microscopy and image analysis permits a more comprehensive measurement of drug responses. For example, Xia and colleagues performed high content screening of over 1,000 pharmacologically-active compounds on lung cancer cells and identified 12 small molecular inhibitors that can suppress drug efflux in these multidrug-resistant cancer cells [132]. Furthermore, imaging-based high-content assays can be applied to 3D cancer spheroid cultures, which better resemble human cancer in small molecule drug screening than do two-dimensional monolayer cultures. Sirenko and colleagues performed screening of 119 approved anti-cancer drugs on colon cancer spheroids and assessed their effects using different morphological parameters, including the number of live and dead cells and the size of the cancer spheroids [133]. With the identification of specific tumor-stroma signaling networks, small molecule inhibitors can be developed through the design of new compounds or by repurposing existing drug compounds for cancer treatment (drug repositioning) [134,135,136].

**CRISPR/Cas9 technology.** The emergence of clustered, regularly interspaced, short palindromic repeat (CRISPR) technology and the generation of the RNA-guided nuclease Cas9 has begun a new era for targeted genome editing in biological research [137]. Guide RNA (gRNA) directs Cas9 nuclease to a specific target DNA site. In the presence of a protospacer adjacent motif sequence immediately following the target sequence, the gRNA/Cas9 complex introduces a double-strand break (DSB). A DSB can be repaired by one of two repair pathways: (1) non-homologous end joining (NHEJ) or (2) homology-directed repair (HDR). DNA repair by NHEJ often results in inserts or deletions that generate frameshifts or premature stop codons while HDR leads to specific nucleotide changes in targeted genes on the basis of the use of a repair template [137,138,139].

Cas9 technology facilitates high-throughput gene editing that utilizes array-based oligonucleotide synthesis generated gRNA libraries. Libraries consisting of ~64,000 to ~87,000 specific gRNAs were used to generate knockout mutations in human and mouse cells for genetic phenotype screening [140,141,142]. To apply the technology *in vivo*, researchers created Cre-dependent Cas9 knock-in mice and injection of adeno-associated virus-, lentivirus-, or particle-mediated gRNA successfully mutated tumor-regulating genes in specific organs *in vivo* [143,144,145]. These encouraging results suggest that CRISPR/Cas9-mediated genome engineering allows the systematic analysis of mutations identified in the cancer genome and facilitates the study of tumor progression. The CRISPR/Cas9 technology is being utilized in research, and a great deal of effort is currently devoted to translating the technology into transformative medical solutions for serious human diseases [146,147]. Further investigation and development of the CRISPR/Cas9 technology in gene editing could provide us with an efficient approach to targeting specific stroma-tumor interactions in combating cancer.

## 6. Conclusions

Despite decades of research, the survival rate of ovarian cancer patients is largely unchanged, and there is a pressing need for identifying novel therapeutic targets in ovarian cancer. Mounting evidence suggests that ovarian cancer progression requires the support of many other cell types within the tumor microenvironment. In this review, we introduced major types of these tumor-supportive stromal cells and highlighted their roles in tumor progression. We also discussed unique stroma-specific crosstalk signaling with malignant cells in ovarian cancer and described multiple approaches that can be used to target the networks identified. Among them, the roles of CAFs in promoting tumor growth, motility, invasion, and other phenotypes associated with poor disease prognosis have recently become a research focus in multiple types of cancer. Compared to the global ablation of tumor stroma, reprogramming CAFs by targeting specific protein/signaling could be developed into a more effective approach for cancer treatment. Multiple studies have shown that ablation of α-smooth muscle actin-positive CAFs in tumors promotes tumor metastasis and reduces survival duration in transgenic mouse models [148,149]. It is believed that changes in the tumor tissue architecture and stroma-tumor interactions caused by such ablation of CAFs could possibly facilitate tumor progression. Taken together the increasing knowledge about ovarian cancer pathogenesis and microenvironment, growing information about CAF-cancer cell signaling networks, and powerful computational modeling tools in integrating and analyzing multiple omics and pathway data, attractive therapeutic targets of CAF-cancer cell crosstalk in inhibiting or regulating ovarian cancer progression would soon be identifiable.
